# Variables Affecting Mortality Among COVID-19 Patients With Lung Involvement Admitted to the Emergency Department

**DOI:** 10.7759/cureus.12559

**Published:** 2021-01-07

**Authors:** Melis Efeoglu Sacak, Sinan Karacabey, Erkman Sanri, Serhad Omercikoglu, Emir Ünal, Özge Ecmel Onur, Haldun Akoglu, Arzu Denizbasi

**Affiliations:** 1 Emergency Medicine, Marmara University Pendik Training and Research Hospital, Istanbul, TUR; 2 Emergency Medicine, Marmara University School of Medicine, Istanbul, TUR

**Keywords:** covid-19, outcomes, mortality

## Abstract

Introduction: A cluster of atypical pneumonia cases in Wuhan, China, turned out to be a highly contagious disease, swept across most of the countries, and soon after was announced as a pandemic. Therefore we aimed to investigate the demographics and factors associated with the disease outcome.

Methods: In this retrospective chart review, we screened patients admitted to the emergency department with severe acute respiratory infection due to coronavirus disease 2019 (COVID-19) between March 15, 2020 and April 30, 2020. Age, gender, symptoms, laboratory data, and radiology data were obtained, as well as outcomes and length of stay.

Results: We identified 177 patients (54.8% male). Seventy-eight percent of the cases were admitted into wards whereas 22% of the cases were admitted into the intensive care unit (ICU). Twenty-five percent of the cases needed invasive mechanical ventilation during their hospital stay and median length of hospital stay until death or discharge was eight days (interquartile range (IQR) 5.0 - 16.0). Among 177 patients, overall in-hospital mortality rate was 19.8% (n=35; male:female=18:17; p=0.6553). In-hospital mortality rates were statistically significantly higher in patients with higher age (64 vs. 74; p=0.0091), respiratory rate (RR) (28 vs. 36; p=0.0002), C-reactive protein (CRP) (54.7 vs. 104.0; p<0.0001), d-dimer (1.2 vs. 3.2; p<0.0001), ferritin (170 vs. 450.4; p<0.0001), fibrinogen (512 vs. 598; p=0.0349), international normalized ratio (INR) (1.1 vs. 1.3; p=0.0001), prothrombin time (PT) (14.8 vs. 17.4; p=0.0001), procalcitonin (0.1 vs. 0.3; p<0.0001), creatinine (0.9 vs. 1.1; p=0.0084), longer length of stay (LOS) (8.0 vs. 13.0; p=0.0251) with lower oxygen saturation (sO_2_) (93.0% vs 87.5%; p<0.0001), diastolic blood pressure (DBP) (78 vs. 70; p=0.0039), lymphocyte (1.2 vs. 0.8; p=0.0136), and with positive polymerase chain reaction (PCR) results (28.6% vs. 12.8%; p=0.0118).

Conclusion: Patients with older age, higher RR, lower sO_2_ and DBP, higher creatinine, d-dimer, INR, CRP, procalcitonin, ferritin, and fibrinogen on initial admission were found to be less likely to survive COVID-19.

## Introduction

In December of 2019, a contagious disease linked with Huanan Seafood Market, causing pneumonia and mostly presenting with fever, cough, fatigue, and occasionally gastroenteritis was reported in Wuhan, China [1]. A novel coronavirus was isolated and it was seen that the virus had 79.5% nucleotide sequence homology to the severe acute respiratory syndrome coronavirus (SARS-CoV), the 2003 SARS outbreak agent [2]. Moreover, as the disease swept across 213 countries, the World Health Organisation (WHO) announced the situation as a pandemic on March 11, 2020, and right after identified the severe acute respiratory syndrome coronavirus 2 (SARS-CoV-2)-induced diseases as coronavirus disease 2019 (COVID-19) [3]. Despite restrictions in most countries, the confirmed number of cases has been rising worldwide. As of today (December 8, 2020), according to WHO data, 66.7 million confirmed cases and 1.54 million deaths from COVID-19 have been reported worldwide [4].

The clinical spectrum of COVID-19 appears to be wide, including mild type without pneumonia, common type with pneumonia, severe type with respiratory deterioration, and critical type with respiratory failure. In addition to acute respiratory failure, the heart, liver, and kidneys may also be involved in the course once the illness has progressed [2,5].

The standard test for detecting COVID-19 is real-time reverse transcription polymerase chain reaction (RT-PCR). A confirmed case of COVID-19 is defined as a positive result on an RT-PCR assay of sputum and throat swab specimens. However, a non-negligible ratio of patients is known to have negative RT-PCR results obtained from pharyngeal swab specimens, although they clinically and radiologically fit the diagnosis. This might be due to not shedding the virus from the upper respiratory tract in the setting of lower respiratory tract involvement, insufficient sensitivity of the testing kits, or improper sampling [2]. 

As a result of incompatibility between RT-PCR and patient clinical course mentioned above, radiological investigation emerged as an easier and faster method to detect patients. Chest computed tomography (CT) scan plays an important role in disease diagnosis, monitoring, severity stratification, and evaluation of treatment response in patients with COVID-19, surpassing the utility of RT-PCR [6]. CT demonstrations of COVID-19 are initially subpleural ground-glass opacity (GGO), and as the disease progresses enlarged GGO with a crazy-paving pattern, followed by extensive bilateral consolidation [7].

On this basis, we aimed to explore the variables for severity of illness in patients with COVID-19 and appeal to clinicians to attach importance to these factors.

## Materials and methods

This retrospective chart and database review study was conducted at a tertiary referral center, as one of the largest among university hospitals with 250,000 adult emergency department (ED) admissions annually. Marmara University School of Medicine - Institutional Ethics Committee approval was obtained (12.06.2020 / 07.2020.678) before initiation of data collection.

As the first COVID-19 cases emerged and were declared on March 10, 2020, health care centers regulated their patient care systems in accordance with the guidelines provided by the Ministry of Health [8]. Accordingly, in our center, patients with suspected COVID-19 were admitted in designated areas. Patients with mild symptoms or symptomless exposure history were examined at an individual zone, whereas patients above 18 years of age with severe acute respiratory infection (SARI) symptoms were admitted into the respiratory red zone designated for possible COVID-19 cases. SARI symptoms are defined as hospitalization necessity due to either dyspnea, tachypnea, hypoxemia, hypotension, or altered mental status in COVID-19-likely patients. If patients were thought to have COVID-19, based on their signs, symptoms, and CT scan, they were referred to infectious diseases for the decision of ward or intensive care unit (ICU) admission. RT-PCR swabs for those patients were obtained immediately after admission, unless obtained and readily available before admission. In the case of negative RT-PCR results, repetitive tests were performed. Patients were included as RT-PCR positive if any of the test results revealed positive.

We retrospectively screened all of the consultation requests from emergency department respiratory red zone to infectious diseases via the Hospital Information System (HIS) to include all consecutive patients admitted to the ED due to SARI symptoms and documented the cases diagnosed with COVID-19 according to the symptoms, clinical manifestations, RT-PCR assay and/or chest CT scans between March 15, 2020 and April 30, 2020. Multiple mottling and GGO with peripheral or subpleural origin and relevant consolidation were reported as “COVID-19 coherent” unless other lung disease findings were prominent in the chest scan. Data regarding the demographics (age and gender), documented symptoms (cough, fever, dyspnea), radiographic findings, laboratory values on admission (white blood cells, neutrophil, leukocyte, platelets, lactate, creatinine, liver function tests, C-reactive protein, procalcitonin, coagulation tests) and obtained at ward or ICU stay (fibrinogen, d-dimer, ferritin) were collected on standard data retrieval forms, if available. With the retrospective nature of the study and after the chart reviews, missing data could not be utilized. We determined the presence of outcomes of the patients (length of stay upon discharge or death, in-hospital death, death in the ED, invasive mechanical ventilation (IMV) requirement, transfer to ICU, admission to a hospital ward, and discharge) and RT-PCR from the HIS and the public health management system (PHMS). Definitions of suspected and confirmed cases, diagnosis, and treatment of COVID-19 was in accordance with interim guidance of the Ministry of Health as well. Data regarding the co-morbid conditions couldn’t be obtained due to the lack of each patient's consent on the electronic information system.

Statistical analysis

Continuous variables were reported as means and standard deviations with their corresponding 95% confidence intervals (CIs, with lower and upper limits), or as medians and interquartile ranges (IQR) according to the distribution pattern of the variables. Comparison of the continuous variables among groups was conducted using a t-test, analysis of variance (ANOVA), Mann-Whitney U or Kruskal-Wallis, according to normality and number of groups. Categorical variables were reported with counts and frequencies and compared with a chi-squared test using contingency tables. The Type 1 error in this study was 5%. We used MedCalc v15.8 (MedCalc Software, Ostend, Belgium) for all statistical analyses.

## Results

We identified 177 patients (54.8% male) who presented to the ED respiratory red zone with SARI symptoms and diagnosed and admitted with possible COVID-19. All patients were treated according to the Ministry of Health Guidelines recommendations as per indicated. Data for invasive mechanical ventilation requirement was missing for five cases. Seventy-eight percent of the cases were admitted into wards whereas 22% of the cases admitted into ICU. Twenty-five percent of the cases needed invasive mechanical ventilation during their hospital stay and median length of hospital stay until death or discharge was eight days (IQR 5.0 - 16.0). Among 177 patients, overall in-hospital mortality rate was 19.8% (n=35; male:female=18:17; p=0.6553). Fifteen patients had neither positive PCR results nor positive CT scan findings. Treatment decision was made depending on their exposure history and symptoms.

Summary of the demographics, symptoms, work-up and laboratory parameters obtained on admission is presented in Table 1 and Table 2. In-hospital mortality rates were statistically significantly higher in patients with higher age (64 vs. 74; p=0.0091), respiratory rate (28 vs. 36; p=0.0002), C-reactive protein (CRP) (54.7 vs. 104.0; p<0.0001), d-dimer (1.2 vs. 3.2; p<0.0001), ferritin (170 vs. 450.4; p<0.0001), fibrinogen (512 vs. 598; p=0.0349), international normalized ratio (INR) (1.1 vs. 1.3; p=0.0001), prothrombin time (PT) (14.8 vs. 17.4; p=0.0001), procalcitonin (0.1 vs. 0.3; p<0.0001), creatinine (0.9 vs. 1.1; p=0.0084), longer length of stay (LOS) (8.0 vs. 13.0; p=0.0251) with lower oxygen saturation (sO2) (93.0% vs 87.5%; p<0.0001), diastolic blood pressure (DBP) (78 vs. 70; p=0.0039), lymphocyte (1.2 vs. 0.8; p=0.0136), and positive PCR results (28.6% vs. 12.8%; p=0.0118). In contrast, there were no differences in systolic blood pressure (SBP), temperature, heart rate (HR), white blood cells (WBC), neutrophil, platelets, aspartate transaminase (AST), alanine transaminase (ALT), activated partial thromboplastin time (aPTT), or lactate between deceased and survivors. Presence of symptoms of cough, fever, and dyspnea on admission were not statistically different between deceased and survivors (p=0.6823, 0.8222, 0.0578; respectively) along with the positive CT signs (p=0.1462), whereas RT-PCR positiveness was significantly higher among deceased (p=0.0118) (chi-square and Mann Whitney tests). 

**Table 1:** Baseline characteristics of patients Percentage refers to the column total. Proportions were compared with Chi-squared test. Medians were compared with Mann-Whitney U test, marked with **, or denoted as P^a^. P-values <.05 are significant. IQR: interquartile range

Demographic Variables	Total (n=177)		Survived (n=142)		Died (n=35)		P		
Age, year; median (IQR)	67.0 (51.0-78.0)		64.0		74.0		0.0091*		
Male sex, n (%)	97 (54.8%)		79 (81.4)		18 (18.6)		0.66		
Tx and Outcome, n (%)									
Intermittent Mandatory Ventilation (IMV) requirement, n (%)	In ED				In-hospital (Overall)				
	12 (6.8%)				43 (25.0%)				
length of stay (LOS), median (IQR)	8.0 (5.0 – 16.0)								
Presenting Symptoms, n (%)	Present	Absent		Total					
Cough	98 (55.7%)	78 (44.3%)		176					
Fever	72 (41.1%)	103 (58.9%)		175					
Dyspnea	127 (72.2%)	49 (27.8%)		176					
Diagnostic Measures	Positive	Negative		Total					
reverse-transcriptase polymerase chain reaction (RT-PCR)	70 (42.7%)	94 (57.3%)		164					
Computed Tomography Scan	147 (86.5%)	23 (13.5%)		170					
Independent Variables	N	Median (IQR)		Survived			Died		P^a^
				N		Median	N	Median	
Age	177	67.0 (51.0-78.0)		142		64.0	35	74.0	0.0091
Systolic Blood Pressure	156	126.0 (108.5 - 147.5)		124		126.5	32	123.5	0.4716
Diastolic Blood Pressure	156	76.0 (65.5 – 86.5)		124		78.0	32	70.0	0.0039
O_2 _Saturation	165	92.0 (87.8 – 96.0)		133		93.0	32	87.5	<0.0001
Body Temperature	146	37.0 (36.5 – 37.9)		119		37.0	27	37.2	0.7236
Heart Rate	136	98.0 (85.0 – 115.0)		111		98.0	25	100.0	0.8397
Respiratory Rate	133	30.0 (24.0 – 36.0)		109		28.0	24	36.0	0.0002
White Blood Cells	175	8.4 (5.9 – 12.3)		140		8.1	35	10.8	0.2225
Neutrophil	175	6.5 (4.0 – 9.7)		139		6.0	35	8.9	0.0587
Lymphocyte	175	1.1 (1.0 – 1.3)		140		1.1	35	0.8	0.0136
Platelets	175	205.0 (148.0 - 263.8)		140		204.5	35	206.0	0.8887
Aspartate Transaminase (AST)	175	38.0 (26.0 – 57.0)		140		37.0	35	40.0	0.4760
Alanine Transaminase (ALT)	175	20.0 (13.0 – 34.0)		140		20.0	35	21.0	0.7020
Creatinine	176	0.9 (0.7 – 1.3)		141		0.91	35	1.0	0.0084
Activated Partial Thromboplastin Time (aPTT)	174	29.9 (27.4 – 32.4)		139		29.9	35	30.4	0.2460
Prothrombin time (PT)	174	15.1 (13.8 – 17.0)		139		14.8	35	17.4	0.0001
International Normalized Ratio (INR)	174	1.1 (1.0 – 1.3)		139		1.09	35	1.3	0.0001
Lactate	165	2.3 (1.6 – 3.4)		131		2.1	34	2.7	0.0672
C-Reactive Protein (CRP)	175	38.0 (26.0 – 57.0)		140		54.7	35	104.0	<0.0001
Procalcitonin	167	0.1 (0.1 – 0.4)		133		0.1	34	0.3	<0.0001
D-dimer	165	1.4 (0.8 – 2.7)		134		1.19	31	3.16	<0.0001
Ferritin	167	211.2 (83.2 – 459.8)		126		170.0	31	450.4	<0.0001
Fibrinogen	135	525.0 (429.0 – 628.5)		105		512.0	30	598.0	0.0349
Length of Stay (LOS)	162	8.0 (5.0 – 16.0)		130		8.0	32	13.0	0.0251

**Table 2:** Comparison of symptoms and work-up regarding in-hospital mortality. Chi-squared test. P-values <.05 are significant. PCR: polymerase chain reaction

Symptoms and work-up		In-hospital mortality					
		Present, n (%)	Absent, n (%)	Total, n (%)			P
Cough	Present	20 (11.4)	78 (44.3)	98 (55.7)			0.6823
	Absent	14 (8.0)	64 (36.4)	78 (44.3)			
	Total	34 (19.3)	142 (80.7)	176 (100.0)			
Fever	Present	13 (7.4)	59 (33.7)		72 (41.1)		0.8212
	Absent	20 (11.4)	83 (47.4)		103 (58.9)		
	Total	33 (18.9)	142 (81.1)		175 (100.0)		
Dyspnea	Present	29 (16.5)	98 (55.7)		127 (72.2)		0.0578
	Absent	5 (2.8)	44 (25.0)		49 (27.8)		
	Total	34 (19.3)	142 (80.7)		176 (100.0)		
CT Scan	Positive	32 (18.8)	115 (67.6)		147 (86.5)		0.1462
	Negative	2 (1.2)	21 (12.4)		23 (13.5)		
	Total	34 (20.0)	136 (80.0)		170 (100.0)		
PCR	Positive	20 (12.2)	50 (30.5)			70 (42.7)	0.0118
	Negative/Inconclusive	12 (7.3)	82 (50.0)			94 (57.3)	
	Total	32 (19.5)	132 (80.5)			164 (100.0)	

## Discussion

The outbreak of COVID-19 has not lost ground since the disease was first identified. As of today, the pandemic is still the leading health concern worldwide. In the presence of this rapidly emerging and remaining situation, identification of variables that could predict disease severity and prognosis on admission is essential to lead clinicians in management. 

In this retrospective study, we further researched the characteristics of hospitalized COVID-19 patients with respiratory symptoms. There were differences in the number in terms of the location, the population, and the season of the epidemic. Among 177 patients, the overall mortality rate was 19.8%, and the median age was 67.0 (IQR: 51.0-78.0). As of December 8, 2020, WHO declared a death ratio of 1.92% for the United States (US), 1.45% for India, 2.68% for Brazil, 1.76% for the Russian Federation, 2.4% for France, 3.48% for Italy, 3.53% for the United Kingdom (UK), and 2.74% for Spain amongst the confirmed cases [4]. Bilinski and Emanuel reported 60.3 COVID-19 deaths per 100.000 for the US, 86.8 for Belgium, 65.0 for Spain, 62.6 for the UK, 59.1 for Italy, 57.4 for Sweden, 46.6 for France, and 36.2 for the Netherlands [9]. In a meta-analysis by Nasiri et al., the mortality rate was reported as 6.6% [10]. As we included the patients with SARI symptoms and indication for hospitalization, our results might not be not comparable with previous reports. In their study, Li et al. included hospitalized patients in Wuhan in a single-center study and reported 27% mortality [7]. As this study has been conducted between January 10, 2020 and February 22, 2020, at the time the disease had newly emerged, this might explain the higher mortality rate than our results. In a study from China, Zhang et al. reported median ages in common, severe, and critical types of disease as 45.0 years (IQR: 35.0 - 55.0), 55.0 years (IQR: 44.0 - 62.0), and 70.0 years (IQR: 55.0 - 73.0), respectively [5]. In our study, we haven’t further stratified the disease stages. Patients with SARI symptoms subtended to the “severe type” and “critical type” cases in Zhang's study. Wu et al. reported that elderly patients had a higher severity rate than younger [2]. Sun et al. included 244 elderly COVID-19 patients in order to define the risk factors for mortality among older adults with COVID-19 and reported that older age was independently associated with risk for death (odds ratio [OR] = 1.122; 95% confidence interval [CI] = 1.007-1.249) [11]. In their study conducted in Wuhan, Pan et al. reported that the median age for non-survivors was significantly higher than the survivors (69.0 vs. 43.0, p<0.001) [12]. Consistent with the previous studies, the median age of the deceased was higher than the survivors in our study (p=0.0091). It is known that as low immunity and comorbidities are seen more commonly with age, these factors may contribute to further physiological deterioration in the setting of illness. 

Male patients consisted of 54.8% of entire patients. There was not a significant difference among the genders regarding mortality (p=0.6553). The male patient ratio is consistent with the US data [13]. In a study conducted in Iran, Raoufi et al. reported no difference between genders in terms of mortality among 380 COVID-19 patients [14]. On the other hand, male gender was reported as a risk factor for COVID-19 severity from previous studies from Asia [2,5,6,12]. This finding is thought to be due to more significant angiotensin-converting enzyme-2 (ACE2) expression reported in Asian men [5]. Other suggestions are that there may be alleles located in ACE2 on the X-chromosome that render resistance to COVID-19 and testosterone has an immunosuppressive effect and moreover, estrogen enhances the immunity [10,15].

Considering initial vital parameters, the median values for SBP, DBP, sO2, body temperature, HR, and RR were 126.0 (108.5 - 147.5), 76.0 (65.5 - 86.5), 92.0 (87.8 - 96.0), 37.0 (36.5 - 37.9), 98.0 (85.0 - 115.0), and 30.0 (24.0 - 36.0), respectively. Compared with survivors, non-survivors had lower DBP (78.0 vs. 70.0; p=0.0039), lower sO2 (93.0 vs. 87.5; p<0.0001), and higher RR (28.0 vs. 36.0; p=0.0002). In another study conducted in our instutituon, body temperature ≥38°C, RR ≥22/min, and sO2 ≤93% were revealed to be related with progression to critical illness among COVID-19 patients [16]. In our study, we recorded the variables as continuous variables instead of categoric, contrary to the study mentioned above. 

In their studies, both Raoufi et al. and Sun et al. reported that the RR and HR were higher (Raoufi et al. p=0.001, p=0.016; Sun et al. p<0.001, p<0.001, respectively), whereas sO2 values were lower (Raoufi et al. p=0.002; Sun et al. p<0.001, respectively) in deceased COVID-19 patients [11,14]. Our findings are mostly consistent with the Sun et al. and Raoufi et al. results. 

In their review, Xie et al. analysed Chinese research focused on the COVID-19 outbreak and extracted 78 records. Xie et al. reported fever (83.0% - 90.9%) and cough (57.6% - 70.8%) as the most common symptoms of the COVID-19 in China [17]. Huang et al. reported fever (80.0%) and dry cough (51.7%) as the leading symptoms among severe COVID-19 patients in Jiangsu province, China [1]. Consistently, a meta-analysis by Nasiri et al. reported fever (83.0%, CI 77.5-87.6), cough (65.2%, CI 58.6-71.2), and dyspnea (27.4%, CI 19.6-35.2) as leading signs and symptoms, respectively [10]. Sun et al. reported fever (86.5%) and dry cough (73.4%) as the leading symptoms among elderly patients with COVID-19 [11]. Raoufi et al. reported cough (60.3%) and fever (55.8%) as the leading clinical manifestations, but found no significance for any of the clinical manifestations between survivors and deceased [14]. In our study, due to including relatively severe patients with SARI symptoms, dyspnea was the leading symptom at presentation (72.2%); yet, similar to the Raoufi et al. study, none of the symptoms of dyspnea, cough, and fever were found to be significantly different between deceased and survivors (p=0.0578, 0.6823, 0.8212, respectively). A finding noticeable in our study is that, among the 33 patients who died in hospital, 13 were admitted with fever complaint whereas 20 denied the symptom on admission. 

Chest CT findings have been an indicator of lung involvement in the disease course. 86.5% of the patients had abnormal CT scan findings, yet, there was no difference between survivors and deceased in terms of abnormal chest CT findings (p=0.1462). Li et al. reported a 98% ratio of abnormal CT scans on admission, but no significant difference between survivors and deceased (p>0.05). Considering the higher mortality rate in their study (27%), higher abnormal CT scan results are consistent with their mortality rate [7]. 

Considering the laboratory values, there was no significant difference between deceased and surviving patients regarding WBC counts, neutrophil counts, and platelet counts in our study. Lower lymphocyte levels were found to be significant for mortality, consistent with Huang's study [1]. Raoufi et al. reported significantly increased WBC and neutrophil counts amongst the deceased COVID-19 patients in Iran, and so did Xie et al. in China [14,17]. Platelet count is known to be independently associated with risk of mortality in ICU, and lower platelet count is involved in various disease severity scores as a risk factor. Also, thrombocytopenia was shown to be associated with three times increased risk of severe COVID-19. This might be either the result of a direct effect on platelets or a systemic inflammation process [18]. Zhao et al. compared the platelet counts on the fifth to sixth day and on the 14th to 15th day between survivors and non-survivors of both genders and found that the mean value of platelet count of survivors was significantly higher than that of non-survivors on the fifth to sixth day (p=0.003) and the 14 to 15th day (p<0.001) of male patients and on the 14 to 15th day (p=0.001) of female patients [19]. In our study, only complete blood counts obtained at time of admission were included. 

Blood lymphocyte levels were significantly lower in the patients who eventually died in hospital (p=0.0136). This finding is consistent with previous reports [5,10,11,14,17,19]. As SARS-CoV is known to mainly act on lymphocytes, for SARS-CoV-2, as a beta-coronavirus, to do the same is not surprising. 

Amongst the acute phase proteins (APP), such as CRP, procalcitonin, ferritin, and fibrinogen, only median CRP values were higher compared to the normal range; yet, as two outcome groups were compared, APPs were significantly higher among deceased than survivors (p<0.0001, <0.0001, <0.0001, =0.0349; respectively). Raoufi et al. reported higher levels of CRP in deceased (89.89 ± 68.71 vs. 46.47 ± 42.77; p<0.0001) than in survivors [14]. Xie et al. reported higher levels of CRP (86%) and serum ferritin (63%) among COVID-19 patients in China, and, consistent with our study, higher levels of D-dimer, serum ferritin, and creatinine in deceased patients [17]. These findings suggest that increased levels of APPs could be indicators for acute respiratory distress syndrome (ARDS) development.

Although lungs are the primary target for the SARS-CoV-2, data suggest that as the disease progresses, with the contributions of the systemic immune inflammation, multiple organ systems are involved in the course of the disease [20]. ACE2 expression has been shown in 2.6% of hepatocytes and 59.7% of cholangiocytes, suggesting that the virus may directly bind cholangiocytes as a target [21,22]. Richardson et al. reported elevated levels of AST and ALT among 5,700 hospitalized COVID-19 patients [23]. In our study, none of the median values for the renal and liver functionality were abnormal on admission, but median serum creatinine level and INR were significantly higher in deceased patients than in survivors (1.0 vs. 0.9; p=0.0084, 1.3 vs. 1.1; p=0.0001). The blood samples for liver and renal damage biomarkers were drawn upon admission. These parameters probably deteriorated as the disease progressed during the hospital stay. Since the blood samples were obtained prior to medication, hepatotoxic effects of the medications administered during the hospital stay were neglected.

Median LOS was significantly longer in non-survivors than in survivors (13.0 vs. 8.0, p=0.0251). US data revealed median LOS of 7.0 [13]. Pan et al. reported longer median LOS in survivors than in non-survivors, with no significant difference (14.0 vs. 18.0, p=0.068) [12]. Our results might be due to early patient discharge in case of signs of improvement in the disease course to maintain the patient circulation and provide healthcare for the mass with limited resources, whereas mandatory extended hospital stay was needed for critical and deteriorating patients.

Limitations

There are several limitations to our study. First, the retrospective design of our study might affect the integrity of data and diminish its credibility, and more prospective cohort studies should be on the agenda in the future. Second, due to the retrospective design and communication challenges with the patients and the next of kin, data regarding the comorbid conditions, pre-existing organ failures, medication, exposure history, time from the exposure to initial symptom onset, and time from initial symptom onset to admission might be quite unreliable. Third, patients enrolled in our study mostly come from Istanbul, and large-scale studies at the national level are needed to provide more reliable and comprehensive data. Fourth, changes in the illness in different subtypes need to be further investigated. A model for predicting the changes of disease is necessary for clinicians to better guide treatments. Fifth, as more than half of the patients were RT-PCR negative, more sensitive testing kits may reveal different results. Last, the treatment protocol is not and has not been standard since the beginning of the pandemic, and there are slight modifications in the therapeutic regimen.

## Conclusions

In conclusion, the retrospective analysis of 177 patients hospitalized with COVID-19 revealed overall mortality of 19.8%. Patients with older age, higher RR, lower sO2 and DBP, higher creatinine, d-dimer, INR, CRP, procalcitonin, ferritin, and fibrinogen on initial admission were found to be less likely to survive COVID-19.
